# Multianalyte Tests for the Early Detection of Cancer: Speedbumps and Barriers

**Published:** 2007-07-10

**Authors:** Michael A. Tainsky, Madhumita Chatterjee, Nancy K. Levin, Sorin Draghici, Judith Abrams

**Affiliations:** 1 Program in Molecular Biology and Genetics, Karmanos Cancer Institute/Wayne State University, 110 E. Warren, Detroit, MI 48201; 2 Department of Computer Science, Wayne State University, 5143 Cass Ave, Room 408 State Hall, Detroit, MI 48202;; 3 Integrated Biostatistics Core, Barbara Ann Karmanos Cancer Institute and Wayne State University, 428 HWCRC, 4100 John R, Detroit, MI 48201

**Keywords:** Complex Diagnostics, Technical Barriers, Regulatory Barriers

## Abstract

It has become very clear that a single molecular event is inadequate to accurately predict the biology (or pathophysiology) of cancer. Furthermore, using any single molecular event as a biomarker for the early detection of malignancy may not comprehensively identify the majority of individuals with that disease. Therefore, the fact that technologies have arisen that can simultaneously detect several, possibly hundreds, of biomarkers has propelled the field towards the development of multianalyte-based *in vitro* diagnostic early detection tests for cancer using body fluids such as serum, plasma, sputum, saliva, or urine. These multianalyte tests may be based on the detection of serum autoantibodies to tumor antigens, the presence of cancer-related proteins in serum, or the presence of tumor-specific genomic changes that appear in plasma as free DNA. The implementation of non-invasive diagnostic approaches to detect early stage cancer may provide the physician with evidence of cancer, but the question arises as to how the information will affect the pathway of clinical intervention. The confirmation of a positive result from an *in vitro* diagnostic cancer test may involve relatively invasive procedures to establish a true cancer diagnosis. If *in vitro* diagnostic tests are proven to be both specific, i.e. rarely produce false positive results due to unrelated conditions, and sufficiently sensitive, i.e. rarely produce false negative results, then such screening tests offer the potential for early detection and personalized therapeutics using multiple disease-related targets with convenient and non-invasive means. Here we discuss the technical and regulatory barriers inherent in development of clinical multianalyte biomarker assays.

## Introduction

The prospects of diagnostic tests for the early detection of cancer using genomic and proteomic technologies have opened a Pandora’s Box of questions on the steps in development of clinical tests. Formatting the reagents into a configuration that is amenable to a clinical laboratory is a rather daunting barrier to a successful clinical test. Even the development of a generic approach to measure 20–100 analytes will require solutions to some unique optimization challenges. Formatting the complete diagnostic test will be more than 20 to 100 times the cost of formatting a single test. A major barrier in multianalyte diagnostics will be the large number of controls and standards required for such a test. To insure tests work properly during development, during production and in customer laboratories, kits require controls and calibrators. This is particularly important for protein-based diagnostics. Controls for this approach may consist of cancer patient sera or potentially panels of human recombinant proteins for each biomarker. Because patient sera containing these biomarkers will be in short supply, the alternative approach of using recombinant proteins for each biomarker is much preferable. However, preparing such recombinant proteins as controls although feasible is clearly a substantial technical challenge. A more practical approach to making a feasible clinical test may be to reduce the number of required biomarkers. Of course, this may not be possible because reducing the number of biomarkers, whether they are serum autoantibodies, circulating proteins, or plasma genomic DNA targets, may result in a test with insufficient accuracy to be clinically useful.

## What is Early Detection?

The clinical utility of an early detection diagnostic approach requires that the temporal development of a positive test result would significantly precede the development of late stage cancer and therefore detect early stage cancer. However there is no established method to define early detection. Moreover, what constitutes early detection may vary among the different types of cancer. For example, the early detection test of an indolent leukemia would have very different requirements from such a test for pancreatic cancer, which often is extremely aggressive at presentation. Different diagnostic settings may require either high specificity or high sensitivity. The prevalence of early stage disease and the costs of false positive and false negative diagnoses must be weighed against the benefits of early diagnosis. In addition, balancing of specificity vs. sensitivity varies with the diagnostic application. For a screening cancer biomarker, one does not want to fail to detect individuals who have early stage but asymptomatic cancer. An ideal screening test would have very high sensitivity, identifying nearly all individuals with disease, but to accomplish that it may falsely identify many individuals who do not have cancer, resulting in lower specificity for the panel of biomarkers, and, subsequently, in unnecessary, invasive, medical testing. Thus it is important that *in vitro* screening tests have both high sensitivity and high specificity. If test sensitivity is valued over test specificity, significant misclassification in the case of low prevalence cancers may result in unnecessary, invasive and expensive medical follow-up. Conversely, valuing specificity over sensitivity may fail to detect cases of cancer. In light of all of these factors, “a one size fits all” approach to diagnostic standards for a multianalyte diagnostic screening test for cancer may be impossible. The balancing of specificity and sensitivity may depend on the nature of the clinical follow-up for each cancer.

## Multianalyte Cancer Diagnostics

Numerous techniques for biomarker discovery have recently emerged for multianalyte diagnostics that are innovative and technically sound. Additional barriers will need to be surmounted for the transition from biomarker discovery to clinically effective tests. These approaches for biomarker discovery can be grouped into *candidate biomarkers* or *undirected searches*. Candidate biomarkers are generally identified in mechanistic studies and then must be found in body fluids such as serum, plasma, saliva, sputum, or urine to be useful. An undirected search begins without any preconceived notion of the identity of the biomarkers in these body fluids and usually involves high throughput screening technology.

## Candidate Biomarkers

A large number of cellular proteins have been shown to be elevated in sera from cancer patients. These proteins are generally referred to as “serum tumor markers” and can be used to monitor disease progression. High levels of various serum biomarkers, for example, CEA and CA19-9, have been reported in different cancers (Nozoe et al. 2006). Intracellular proteins such as CA125 may be released by dying cells and detected in serum (Beck et al. 1998). Furthermore, serum is the storehouse for autoantibodies that are produced due to the activation of humoral immune response against tumor associated antigens, and indeed, several reports are based on the study of tumor infiltrating lymphocytes in tumors which are the root cause for generation of anti-tumor immune response (Matsutani et al. 2004). It is well established that DNA levels are elevated in plasma from cancer patients. Plasma DNA is derived either from tumor apoptosis (Lichtenstein et al. 2001) or from secretion of DNA by cells (Stroun et al. 2000). Saliva which bathes mucosa from oral cavity to larynx is an efficient protective medium- an antibacterial, antiviral, antioxidative, etc. Tests using saliva as a diagnostic tool have been used in clinical and research areas. The presence of tumor markers, CA15-3 and c-*erb*B-2, Cathepsin-D and p53 in saliva of women with breast carcinoma have been reported (Streckfus et al. 2000). Saliva has been also used for oral diagnostics to detect antibodies to HIV (Malamud et al. 1997). Sputum samples from patients are clinically useful for the detection of lung cancer. As discussed earlier, cigarette smoking can lead to a series of genetic alterations and is the major cause of the development of lung cancer. Sputum cytology is a noninvasive test and is widely used as a diagnostic test for bronchogenic carcinoma (Murray et al. 2002). Urine samples from patients are tested for the detection of urinary bladder cancer. Urine has large collection of tumor cells and a diagnostic test for the detection of urothelial carcinoma can easily be achieved by flow cytometry (Cunderlikova et al. 2007).

A number of cancer-specific genomic events have shown potential for blood-based early detection diagnostics. These cancer-specific genomic events include gene translocations (Shimazaki et al. 1997; Tomlins et al. 2005), point mutations by PCR-based sequencing (Hibi et al. 1998; Jeronimo et al. 2001), DNA methylation using sequence analysis of bisulfite treated plasma DNA (Belinsky et al. 2005; Esteller et al. 1999) or the appearance of novel RNA molecules by RT-PCR (Calin and Croce, 2006). These analyses are dependent on the shedding of nucleic acids from tumor cells into the general circulation. There has been amazing progress towards the development of clinically acceptable high-throughput techniques for the detection of such genomic changes for the early detection of cancer. These techniques often indicate the presence or absence of a detectable genetic event but the diagnostic indications of any single cancer-specific genomic event must be determined individually. These approaches are often derived from candidate biomarkers identified from mechanistic studies of cancer development. From those mechanistic studies the diagnostic limitations and ramifications of such genomics biomarkers may be apparent but their utility will have to be demonstrated in a screening setting. In spite of this limitation, the etiologic relationship to the cancer makes such biomarkers quite compelling.

## Undirected Approaches to Biomarker Discovery

An early undirected approach to the discovery of diagnostically useful tumor antigens referred to as SEREX (serological expression cloning of recombinant cDNA libraries of human tumors) was introduced by Sahin, et al. for identifying human tumor antigens eliciting a humoral immune response (Sahin et al. 1995). This technology was designed to isolate tumor antigens that have elicited high-titer IgG responses in human hosts. Briefly, cDNA libraries are constructed from a fresh tumor specimen. The cDNA phage library is plated, transferred to nitrocellulose membranes which are then immunoscreened with autologous patient’s serum. This type of procedure of screening the cDNA expression library is quite laborious and requires large number of membrane filters blotted with bacteriophage plaques, which are then screened with sera from cancer patients, usually available in small quantities. This technology has been used for the detection of relevant tumor antigens eliciting humoral immune response in many different cancers. Overexpression of normal gene products or mutation in cancer may be a major underlying mechanism for the immunogenicity of cancer antigens in cancer patients. SEREX-defined antigens, MAGE and tyrosinase, have been detected in melanoma patient sera (Stockert el al. 1998). High antibody response to NY-ESO-1 has been detected in stage IV melanoma patients (Stockert el al. 1998). A classic example of mutational antigen is the tumor suppressor gene p53, which has been identified by SEREX of ovarian cancer (Stone et al. 2003). Several antigens for example, eIF-4 gamma in lung cancer (Brass et al. 1997) and HER-2/*neu* in breast cancer (Scanlan et al. 2001), which are overexpressed and have mounted an immune response, have been identified by SEREX. Although SEREX has been successful in identifying tumor antigens, this technology tends to identify antigens that were overexpressed in the tumor used in the discovery steps and not many other patients, possibly because an autologous patient’s serum was used for immunoscreening of tumor cDNA libraries.

For high throughput antigen cloning, our group has developed an undirected approach using phage display techniques (“differential biopanning”) to identify the cancer antigen space within the human proteome (Chatterjee et al. 2006; Draghici et al. 2005) Differential biopanning involves immuno-screening of T7 phage tumor-derived cDNA libraries using a 2-step process, starting with serum IgGs pooled from different age-matched normal healthy individuals. This step helps in the removal of non-tumor/common antigens that bind to IgGs in normal sera. Next, serum IgGs from cancer patients are used as the bait in biopanning in order to enrich for clones of tumor antigens. The bound antigens are eluted and the resulting phage clones are amplified for the next round of biopanning. Generally after four cycles of biopanning, phage clones are picked from multiple independent patients and then robotically printed on protein microarrays to identify circulating serum antibodies produced by the cancer patient presumably to the cancer cells or tissue. Microarrays are processed with several sera obtained from cancer patients as well as healthy individuals. Those arrays are then further processed with Cy3 labeled T7 monoclonal antibody, directed against phage capsid protein, and Cy5 labeled goat anti-human IgG that recognizes the test subject’s IgG bound to the antigens on the arrays.

After processing, arrays are scanned and the ratio of anti-T7 capsid and anti-human IgG is determined by comparing the fluorescence intensities in the Cy3- and Cy5-specific channels at each spot using standard image analysis programs. Statistical analysis is performed on the dataset of these dye ratios and is further validated using an independent set of patients and controls (Draghici et al. 2005; Chatterjee et al. 2006).

The top ranking antigens obtained from statistical analysis are readily amenable to reformatting into an immunoassay as a diagnostic predictor of cancer. The utilization of this diagnostic test in the clinic would be as a periodic, *in vitro* diagnostic screening immunoassay to detect the presence of cancer. This approach has been adopted by others in the field of antigen biomarkers (Wang et al. 2005; Zhong et al. 2005). The ultimate goal would be to therapeutically intervene via personalized immunotherapy very early in the development of the cancer.

In recent years a number of serum proteomics approaches have sought to identify circulating proteins that are indicative of the presence of cancer in the test subject. The identification of tumor-specific overexpressed proteins is often performed by analyzing RNA (Miura et al. 2005) and then testing whether those proteins are indeed present in the serum or plasma proteomes. Their study has shown that human telomerase reverse transcriptase (hTERT) mRNA has higher expression in patients with hepatocellular carcinoma (HCC) than those with chronic liver diseases. Many interesting biomarkers are lost at this point because those upregulated RNAs do not result in increased proteins in body fluids or tissues.

In an alternative approach to directly study protein expression in body fluids or tumor tissues (Psyrri et al. 2007) a novel method of compartmentalized was developed for *in situ* protein analysis so as to determine the prognostic value of the p53 biomarker in ovarian cancer. This approach was based on the construction of tumor tissue microarray from each patient. The tissue core was obtained from paraffin embedded tissue blocks and immunohistochemical staining was performed (Psyrri et al. 2007) with the tissue microarray slides utilizing the antibody which recognizes both wild type and mutant p53. Their study correlated the higher p53 expression with better outcome for overall survival at 5 years. An effective diagnostic test will require panels of such biomarkers, however only a fraction will indeed be present in the serum of cancer patients and not healthy test subjects. An additional barrier to the development of a diagnostic test for each protein is the reformatting the assay into the form of a sandwich ELISA. ELISA assays require one antibody (the “capture” antibody) that is immobilized to a solid phase attached to the bottom of a plate well. Antigen is then added which forms a complex with the capture antibody. The unbound products are then removed with a wash, and a labeled second antibody (the “detection” antibody) is allowed to bind to the antigen, thus completing the “sandwich”. The assay is then quantitated by measuring the amount of labeled second antibody bound to the matrix, through the use of a colorimetric substrate. Many otherwise good biomarkers fail at this step of development because of the difficulty of developing antibody pairs for a sandwich ELISA test. This barrier is compounded when a specific panel of protein biomarkers is needed to proceed to develop the final test and loss of any single member of the panel lowers the accuracy to unacceptable levels. An alternative approach is to begin with existing pairs of antibodies to candidate biomarker proteins that perform well in an ELISA diagnostic and then determine whether any of these proteins are informative biomarkers for the early detection of cancer. However, a significant barrier exists in the enormous range of concentrations of proteins in blood or other body fluids and parallelizing such tests on a single platform.

A fairly recent technologic platform referred to as Luminex xMAP (Jones et al. 2002) has been reported that permits, in principle, multiplexing of up to 100 analytes in a single reaction and is suited to a wide range of applications such as profiling using immunodiagnostics for candidate serum proteins or antibodies. The Luminex technology is based on color coding tiny beads, called microspheres, into 100 distinct sets. Each bead set can be coated with a reagent specific to a particular bioassay, allowing the capture and detection of specific analytes from a sample. Within the Luminex analyzer, lasers excite the internal dyes that identify each microsphere particle, and also any reporter dye captured during the assay. Many readings are made on each bead set, permitting robust statistical analysis of the results. The bead-based suspension array technology allows simultaneous analysis of antibodies with specificities for up to 100 different antigens in a single reaction. In an ELISA similarly formatted for multianalyte testing, the reactivity of one serum to several antigens requires individual reactions, resulting in high serum consumption. While overcoming this challenge, Luminex technology is limited by the fact that all antibodies present in human sera can directly bind to the beads, with the potential of non-specific background (Waterboer et al. 2006). This technology has been applied to determine angiogenic profiles in the plasma of nude mice bearing human tumors (Keyes et al. 2003) and for the detection of increased levels of cytokines in cancer patients (Gorelik et al. 2005).

A serum proteomic technology based on the generation of proteomic spectra of serum proteins using matrix-assisted laser desorption and ionization time-of-flight (MALDI-TOF) and surface-enhanced laser desorption and ionization time-offlight (SELDI-TOF) has been reported by Petricoin et al. for diagnosis of ovarian cancer at early stage (Petricoin et al. 2002). In MALDI-TOF MS, a small amount of specimen containing peptides and protein (1 μl) is dried on a target plate together with a light-absorbing matrix molecule. The mixture of protein and matrix from surface deposits is vaporized by nanosecond-duration laser pulses. This results in the release of ionized protein molecules, which are accelerated in an electric field within a vacuum. Ions with low mass/charge ratios (*m/z*) are accelerated to higher velocities and reach the detector before ions with a high *m/z.* The basic principle between MALDI-TOF MS and SELDI-TOF MS is the same except in SELDI-TOF MS, retentate chromatography is performed directly on the surface of the target plate. The target surface (chip) is modified to contain ion-exchange, hydrophobic, normal-phase, or metal chelate functional groups, and proteins are selectively captured on the surface depending on the protein properties and selection of binding and wash buffers. When Petricoin et al. applied this SELDI to a specific population of ovarian cancer patients, this technology resulted in a specificity of 95%. Despite its initial, apparent success, this technology has faced serious concerns regarding the reproducibility of the data and the artifacts in sample preparation, storage and processing which may have biased the data (Diamandis, 2004).

Although not for early detection of cancer, a new prognostic test that has been developed by scientists at the Netherlands Cancer Institute for evaluation of the recurrence of breast cancers is referred to as MammaPrint^®^ (van’t Veer et al. 2002; van de Vijver et al. 2002). MammaPrint^®^ is a DNA microarray-based *in vitro* diagnostic test that provides information about the probability of tumor recurrence by measuring the expression of 70 genes previously identified in a large undirected survey of breast cancer tissues. The MammaPrint^®^ test measures the expression level of each of these genes in a specimen of a woman’s surgically-removed breast cancer tumor and then uses a specific algorithm for the generation of a score that determines whether the patient is considered to be low risk or high risk for the spread of tumor cells (van’t Veer et al. 2002). The study of van de Vijver et al. revealed that the expression profile of 70 genes when tested in a series of 295 consecutive breast cancer patients (most of them received adjuvant chemotherapy or hormonal therapy at the hospital of Netherlands Cancer Institute) performed best as a predictor of appearance of distant metastases during first five years after treatment (van’t Veer et al. 2002). MammaPrint^®^ is marketed by Agendia BV (Amsterdam, The Netherlands) was the first multianalyte test to obtain approval from FDA to predict breast cancer recurrence. MammaPrint^®^ has provided a better understanding for the process of approval of *in vitro* multianalyte diagnostic tests.

## Translating Bioinformatics Techniques into Clinically Applicable Algorithms

There are a number of bioinformatics and biostatistical techniques for performing undirected searches to identify a set of biomarkers that accurately identify individuals with cancer and those without. In our laboratory we have primarily used neural networks to identify biomarkers that accurately classify individuals as positive or negative for specific cancers, but we are also investigating the use of random forests. No matter what technique is used, the results must be scrupulously validated using cross-validation or bootstrapping techniques to ensure that the technique has not overfitted the data. Unfortunately, the accuracy thresholds necessary for a diagnostic test cannot be chosen independently of the application. The application depends on the disease prevalence and the costs of a false positive and false negative. For instance, if the cost of a false positive is minimal, high sensitivity is to be preferred. If the cost of a false positive is high, high specificity is desirable. The exact balance depends on the disease, its prevalence, the costs of follow-up exams and the risks of not detecting the illness. Currently there is no federal guidance for diagnostic test performance for a disease in which there is no existing diagnostic test. The performance characteristics must result from a test employing a fixed set of analytes detecting the disease. Variations in the algorithms or the set of analytes (biomarkers) will require recertification of the diagnostic test.

## Regulatory Issues

While the F.D.A. regulates diagnostic tests sold to laboratories, hospitals and physicians, it historically allowed tests developed by and performed in a single laboratory to be offered without F.D.A. approval. These labs are regulated by the federal Medicare agency under the Clinical Laboratory Improvement Amendments of 1988. However, in September 2006, the F.D.A. announced that it intended to require approval for “home brew” tests that examine multiple genes or proteins and use an algorithm to compute a result. To that end the F.D.A. issued a draft guidance document for *In Vitro* Diagnostic Multivariate Index Assays (IVDMIAs). IVDMIAs are defined as “test systems that employ data derived in part from one or more *in vitro* assays, and an algorithm that…runs on software to generate a result that diagnoses a disease or condition or is used in the cure, mitigation, treatment, or prevention of disease.” The guidance document addresses the need for these IVDMIAs to meet pre-market review and post-market device requirements. On February 6, 2007, for the first time, the F.D.A. cleared the marketing of an IVDMIA that profiles genetic activity for the purpose of predicting breast cancer recurrence, MammaPrint^®^. (New York Times, 2007;U.S. Food and Drug Administration, 2006a; U.S. Food and Drug Administration, 2006b).

## Assay Validation

A number of further barriers await those who produce a clinical diagnostic multianalyte test for cancer. A good sense of these barriers is evident by contrast to single analyte tests. There are two major sources of inter-test variation, the analyte and the assay reagents. For a single analyte test, once the stability characteristics in the body fluid are established, a standard operating procedure that can yield consistent results should emerge. However, if each member of the panel of analytes has different stability characteristics in the body fluid being tested, then there is a greater risk of inter-analyte and inter-laboratory variation. Similarly, if the specific components of the test system have differing stability in their clinical test format, the different components may have varying shelf lives and these challenges increase geometrically with the number of analytes. There is also the possibility of assay interference among analytes. Ideally cases and controls should be accrued in a manner similar to the clinical implementation such as an asymptomatic population who later developed cancer but not know it at the time of sample donation. Such a cohort is the gold standard for early detection assay validation. Lastly few clinical diagnostic platforms for proteins are available that can accommodate 20–100 biomarkers in a parallel assay format. While such technologies are undoubtedly under development, they will surely emerge in the diagnostic marketplace with some conservative applications rather than for the validation of a novel panel of cancer biomarkers. A natural question is how soon after that initial application will cancer biomarkers panels be evaluated? That will probably depend on the feasibility of widespread implementation of such novel platforms, the clarity of clinical and regulatory pathways for their use, and the ability to acquire sufficient reimbursement for their increased complexity. These new tests will require some new premarket pathways and postmarket requirements.
